# Evaluation of ultraviolet‐C and spray‐drying processes as two independent inactivation steps on enterotoxigenic *Escherichia coli* K88 and K99 strains inoculated in fresh unconcentrated porcine plasma

**DOI:** 10.1111/lam.13068

**Published:** 2018-09-19

**Authors:** E. Blázquez, C. Rodríguez, J. Ródenas, A. Pérez de Rozas, J.M. Campbell, J. Segalés, J. Pujols, J. Polo

**Affiliations:** ^1^ APC EUROPE, S.L.U. Avda Granollers Spain; ^2^ IRTA Centre de Recerca en Sanitat Animal (CReSA, IRTA‐UAB) Campus de la Universitat Autònoma de Barcelona Bellaterra, Barcelona Spain; ^3^ APC Inc. Ankeny IA USA; ^4^ Departament de Sanitat i Anatomia Animals Universitat Autònoma de Barcelona (UAB) Bellaterra, Barcelona Spain; ^5^ UAB Centre de Recerca en Sanitat Animal (CReSA IRTA‐UAB) Campus de la Universitat Autònoma de Barcelona Bellaterra, Barcelona Spain

**Keywords:** blood derivatives, *Escherichia coli*, porcine plasma, spray‐drying, ultraviolet irradiation

## Abstract

The objectives of this study were to assess the effectiveness of an ultraviolet (UV‐C, 254 nm) irradiation system and the spray‐drying method as two independent safety steps on inactivation of *Escherichia coli* K88 and K99 spiked in porcine plasma at 6·46 ± 0·04 log_10_ ml^−1^ and 6·78 ± 0·67 log_10_ ml^−1^ respectively for UV‐C method, and at 7·31 ± 0·39 log_10_ ml^−1^ and 7·66 ± 0·11 log_10_ ml^−1^, respectively for the spray‐drying method. The UV‐C method was performed at different UV light doses (from 750 to 9000 J l^−1^) using a pilot plant UV‐C device working under turbulent flow. Spray‐drying treatment was done at inlet temperature 220 ± 1°C and two different outlet temperatures, 80 ± 1°C or 70 ± 1°C. Results indicated that UV‐C treatment induced a 4 log_10_ viability reduction for both *E. coli* at 3000 J l^−1^. Full inactivation of both *E. coli* strains was achieved in all spray‐dried samples dehydrated at both outlet temperatures. The special UV‐C system design for turbid liquid porcine plasma is a novel treatment that can provide an additional redundant biosafety feature that can be incorporated into the manufacturing process for spray‐dried animal plasma.

**Significance and Impact of the Study:**

The safety of raw materials from animal origin such as spray‐dried porcine plasma (SDPP) may be a concern for the swine industry. Ultraviolet treatment at 254 nm (UV‐C) of liquid plasma has been proposed as an additional biosafety feature in the manufacturing process of SDPP. We found that UV‐C exposure in the liquid plasma at 3000 J l^−1^ reduces about 4 log10 ml^−1^ for *E. coli* K88 and K99. Full inactivation of both *E. coli* strains was achieved in all spray‐dried samples. The incorporation of UV‐C treatment to liquid plasma improves the robustness of the SDPP manufacturing process.

## Graphical abstract

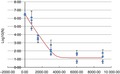

## Introduction

Spray‐dried animal plasma (SDP) is a protein source extensively used in pig feed due to its functional components that contribute to improved post‐weaning performance and survival (Torrallardona 2010). However, the safety of raw materials from animal origins is a concern for the swine industry. Ultraviolet (UV) treatment of liquid plasma has been proposed to introduce an additional redundant inactivation step in the manufacturing process of SDP to further enhance biosafety of the final spray‐dried product (Polo *et al*. 2015; Blázquez *et al*. 2017).

Ultraviolet exposure at a wavelength of 254 nm (UV‐C) is a nonthermal process that has a germicidal effect by causing thymine‐thymine and thymine‐cytosine dimers in DNA and thymine‐uracil dimers in RNA, which disrupts microbial reproduction (Jagger 1967). During the spray‐drying process, thermal inactivation, high pressure and rapid dehydration are the phenomena involved in microbial inactivation. Although the most important site of damage caused by dehydration is the cytoplasmic membrane (Crowe *et al*. 1987; Lievense & van 't Riet 1994; Perdana *et al*. 2013; Huang *et al*. 2017), dehydration also produces damage to DNA/RNA and protein (Lievense 1992). Thus, the sequential action of both methods for the plasma production process is promising to inactivate micro‐organisms, since both damage different targets involved in microbial inactivation.

Enterotoxigenic *Escherichia coli* is one of the main causes of enteric disease and death in newborn and weaned pigs (David 2002) and is the major cause of neonatal diarrhoea in calves (Acres 1985). *E. coli* requires the expression of adhesion fimbriae (adhesins), which are encoded in plasmids, to be adhered to the intestinal epithelium. *E. coli* expressing K88 adhesin is mainly found in pigs (Gaastra and De Graaf 1982), while K99 is the main adhesion antigen found in bovine species (Tzipori 1981), although K99 can also be found in ovine and porcine species (Gaastra and De Graaf 1982).

The aim of this study was to assess the effectiveness of a UV‐C treatment system on *E. coli* inactivation after inoculation in fresh unconcentrated liquid porcine plasma. In addition, a second objective was to test the effectiveness of the spray‐drying process on the inactivation of *E. coli* at two different outlet temperatures, at the regular outlet temperature normally used by the industry (80°C) and at lower outlet temperature (70°C).

## Results and discussion

### UV‐C test

**Figure 1:**
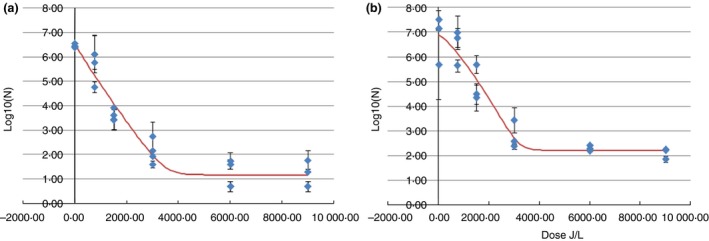
Inactivation kinetics of the strains *Escherichia coli* K88 (a) and *E. coli* K99 (b) after UV‐C irradiation. *Escherichia coli* K88 presented a Log linear plus tail inactivation kinetics while *E. coli* K99 showed a Weibull plus tail inactivation kinetics. ‘Measured’ indicate the real data obtained during the experiment. ‘Identified’ is the best model fit for prediction kinetics obtained by the GInaFiT program 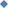 Measured ; 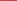 Identified [Colour figure can be viewed at http://wileyonlinelibrary.com]

Plasma inoculated with *E.coli* K88 strain had an initial count of 6·46 ± 0·04 log_10_ ml^−1^. After UV‐C treatment at 3000 J l^−1^, bacterial counts showed a significant reduction of 4·34 log, describing a curve adjusted to the log linear plus tail model (Fig. 1) with a regression coefficient of *R* ^2^ = 0·95 (Table 1). At doses of 6000 and 9000 J l^−1^, residual *E. coli* populations of 1·18 ± 0·30 and 1·12 ± 0·30 log_10_ ml^−1^ were counted, respectively. The UV‐C doses required to have 4 log_10_ reduction (log10R) was predicted to be 3105 J l^−1^.

**Table 1:** Statistical parameters of the two models for inactivation applied to results obtained with strains *Escherichia coli* K88 *and* K99

	*E. coli* K88		*E. coli* K99	
	Log linear plus tail	Weibull plus tail	Log linear plus tail	Weibull plus tail
MSE^a^	0·2594	0·2686	0·3874	0·3835
RMSE^b^	**0·5093**	0·5182	0·6224	**0·6193**
R‐square	0·9504	0·9511	0·9167	0·9235
R‐square adjusted	0·9457	0·9438	0·9048	0·9058
4D reduction^c^ reached at (J l^−1^)	3105·9	3105·9	3427·2	3427·2

a MSE: Mean sum of squared error.

b RMSE: Root mean sum of squared error. The lowest RMSE value determines the inactivation model with the best fit. The lowest RMSE value is showed in bold in the table.

c 4D reduction: UV‐C dose irradiation in J l^−1^ at which a 4 Log reduction was achieved.

Plasma inoculated with the strain *E. coli* K99 had an initial count of 6·78 ± 0·67 log_10_ ml^−1^. After UV‐C treatment, bacterial counts decreased significantly, showing a curve adjusted with the Weibull plus tail model, with a regression coefficient of *R* ^2 ^= 0·923 (Table 1). There was a 3·97 log_10 _ml^−1^ reduction of the initial count between 0 and 3000 J l^−1^ (Fig. 1). Residual populations of 2·30 ± 0·08 and 2·11 ± 0·15 log_10 _ml^−1^ were counted after irradiation at doses of 6000 and 9000 J l^−1^, respectively. The 4 log10R was predicted to be achieved at 3427 J l^−1^.

### Spray‐drying test

Full inactivation of strains *E. coli* K88 and K99 was achieved in all spray‐dried samples dehydrated at an inlet temperature of 220 ± 1°C and both outlet temperatures of 80 ± 1°C or 70 ± 1°C (Table 2).

**Table 2:** Effect of the outlet temperature on the inactivation of each *Escherichia coli* strain tested

	*E. coli* K88 strain (CFU log_10_ g^−1^ solids) ± SD	*E. coli* K99 strain (CFU log_10_ g^−1^ solids) ± SD
80°C outlet air temperature		
Inoculated plasma	7·31 ± 0·39	7·66 ± 0·11
Spray‐dried plasma (SDP)	<1	<1
70°C Outlet air temperature		
Inoculated plasma	6·93 ± 0·5	7·44 ± 0·42
SDP	<1	<1

Numerous studies have demonstrated the effectiveness of the spray‐drying process used during the manufacturing of SDP, providing evidence that SDP is a biologically safe product relative to multiple pathogens of concern for the swine industry (Polo *et al*. 2005; Pujols *et al*. 2007, 2008, 2014; Gerber *et al*. 2014). However, it is prudent to evaluate additional biosafety features that may further enhance the robustness of the SDP production process. Exposure to UV‐C is extensively used for the disinfection of liquid media and surfaces due to its germicidal activity (Guerrero‐Beltran 2004; Lin *et al*. 2012). Previous research has demonstrated that UV‐C treatment of liquid plasma was effective to inactivate *Porcine parvovirus* (Polo *et al*. 2015) and *Salmonella* spp. (Blázquez *et al*. 2017) inoculated in liquid plasma. During the spray‐drying process, temperature and dehydration are the mechanisms that contribute to microbial mortality (Perdana *et al*. 2013; Huang *et al*. 2017), whereas, UV‐C treatment causes damage to nucleic acids (Jagger 1967) and protein‐DNA cross links (Smith 1962).

In this study, UV‐C inactivation kinetics of two strains of *E. coli* from porcine (K88) and bovine (K99) origins were very similar, although such kinetics fit better to different models, as indicated by the lower RMSE in each case. For both strains of *E. coli*, a rapid decrease in bacterial count was observed between 0 and 3000 J l^−1^ of UV‐C, with the appearance of a residual population (Nres) afterwards. These results agree with other UV‐C inactivation studies performed with *E. coli* (Hijnen 2006). The reduction of the inactivation rate at high UV fluencies (tailing) could be caused by micro‐organism aggregation, appearance of a resistant subpopulation, hydraulic design (Hijnen 2006), matrix effect or particle size effect (Winward 2008). Porcine plasma is a dense, coloured, liquid matrix with 8–10% solids, and contains a complex blend of different proteins with some of the proteins having binding properties (Burnouf 2007). Therefore, the matrix and particle size effects of porcine plasma may have had a special impact on the tailing effects of UV‐C treatment in the present study.

The residual population of *E. coli* after UV‐C treatment should apparently be eliminated in the subsequent spray‐drying process based on the total inactivation results by the spray‐drying methods at the two outlet temperatures tested (Table 2). The outlet spray‐drying temperature is 80°C for commercial manufacturing of SDP (Pérez‐Bosque *et al*. 2016) and results of the present study suggest that both *E. coli* strains are very susceptible to spray‐drying even at a lower outlet temperature (70°C). These results provide confidence that current commercial spray‐drying conditions are highly effective for inactivation of *E. coli*.

Processing steps should be able to remove or inactivate a wide range of pathogens, according to the World Health Organization (WHO, 2004) guidelines on viral inactivation and removal procedures intended to assure the viral safety of human blood plasma products. These guidelines recommend that two or more robust, effective and reliable processes will be able to remove or inactivate 4 logs or more of viruses. Although the inactivation of viruses has to be considered separately, these guidelines used for virus safety in human plasma transfusion products can be applied to pathogens in general that affect animal blood then UV‐C light treatment at 3000 J l^−1^ and spray‐drying can be considered two robust safety procedures in the production of SDP since both methods individually inactivated at least 4 log_10_ *E. coli*.

In addition, the manufacturing process of SDP has other safety features, such as blood collection from healthy animals declared fit at slaughter for human consumption, pooling of inherent neutralizing antibodies (NA) against potential pathogens, and post‐packaging storage in a dry environment at room temperature for at least 14 days (Pérez‐Bosque *et al*. 2016). Plasma pooling is also a recognized safety step in the production of certain human plasma products (Solheim *et al*. 2000, 2006, 2008), since there is successful neutralization of antigens in the presence of NA. Some micro‐organisms in dehydrated form and stored under appropriate constant conditions can remain viable in a unique vitrified state for very long times, even years (Perdana *et al*. 2013). Spray‐dried plasma has a water activity of <0·6 and is packaged and stored in mild temperatures. The storage conditions for SDP held at room temperature (*c*. 20°C) for 14 days has been demonstrated effective to inactivate *Porcine epidemic diarrhea virus*,* Porcine reproductive and respiratory syndrome virus* and other coronaviruses when these viruses were experimentally inoculated on spray‐dried plasma (Pujols and Segalés 2014; Sampedro *et al*. 2015). However, it is unknown if these storage conditions affect *E. coli* or other bacteria survival in spray‐dried plasma. In the present study, the SDP storage temperature effect (20°C for 14 days) on *E. coli* survival was not tested because both *E. coli* strains did not survive the spray‐drying process. All the above‐mentioned safety features involved in the manufacturing process of SDP use different inactivation mechanisms, and collectively ensure the biosafety of SDP.

In conclusion, this study provides evidence that affordable levels of UV‐C treatment (3000 J l^−1^) of liquid porcine plasma can significantly decrease *E. coli* bacterial counts (4 log_10_ ml^−1^ at 3000 J l^−1^). Furthermore, the study indicated that both UV‐C treatment and spray‐drying as independent safety procedures are very effective for inactivating *E. coli* K88 and K99. This novel UV‐C technology can be adapted to further enhance the robustness of the manufacturing process for assuring the biosafety of spray‐dried plasma.

## Materials and methods

### Bacterial strains and test products

Two strains of *E. coli* were used in the present study: an isolate from swine expressing the K88 adhesin, and a second isolate from bovine expressing the K99 adhesin (both isolates were kindly provided by Dr. Antonio Juárez. University of Barcelona, Spain). A 0·3 ml volume of *E. coli* isolates was cultured in 100 ml of LB media (Sigma‐Aldrich) at 37°C and 150 rev min^−1^ for 18 h. The cells were subsequently concentrated by centrifuging (1000*g* for 20 min at 4°C) using sterilized 40 ml tubes containing 20 ml of culture media. The remaining culture media was removed by resuspending the cell precipitate in 20 ml of sterile 0·01 g mol^−1^ phosphate buffer saline (PBS). After resuspension, it was centrifuged again as described above and the resulting cell precipitate that was resuspended again in 500 ml of PBS reaching a final titre of 8·98 log_10_ CFU per ml for K88 and 8·91 log_10_ CFU per ml for K99 .

Fresh liquid porcine plasma from the production plant of APC Europe S.A., (Granollers, Spain) was used for these trials. This plasma was obtained by centrifugation of blood from pigs processed at a local officially inspected abattoir.

### Settings of pilot scale UV‐C system

The UV‐C reactor system (SP1) was designed and manufactured by Sure Pure Operation AG (Zug, Switzerland) that has already been described by Blázquez *et al*. (2017). The configuration of the pilot scale UV‐C reactor consisted of a closed system with one low pressure mercury UV lamp (30 UV‐C Watts, 254 nm) surrounded by a quartz crystal. The plasma flowed through a steel tube containing a vortex (internal grooved spiral tube that generated a turbulent flow) between the spiral tube and the quartz sleeve. The tangential inlet of the reactor created high velocity and turbulence in the inlet chamber improving liquid contact with the UV‐C light. The liquid was pumped from the inlet chamber into the reactor at a constant flow rate of 4000 l h^−1^ to achieve a Reynolds value greater than 2800 which is indicative of a turbulent flow (Simmons *et al*. 2012). Plasma was pumped from the tank to the UV‐C lamp and recirculated many times through this circuit to achieve the required UV‐C dose *vs* time. The time spent by the liquid to pass through the system once was 7·2 s, delivering 22·95 J l^−1^ or 23·40 mJ cm^−2^ per cycle.

### UV‐C test

A total of 60 kg of plasma were used for the present study, 30 kg for each of the tested *E. coli* strains. For each isolate, the 30‐kg batch was divided into three 10‐kg sub‐batches to conduct tests in triplicate. Because liquid fresh plasma from the abattoir may contain different micro‐organisms, the initial 60 kg of plasma product was treated by UV‐C at 10 000 J l^−1^ for 1 h to inactivate any potential bacteria prior to artificial inoculation with *E. coli*.

Plasma was spiked with an inoculum of 90 ml of *E. coli* K88 (ratio 1/330) and 220 ml in the case of *E. coli* K99 (ratio 1/138). After inoculation, the liquid was recirculated through the UV‐C device for 3 min before activating the UV lamp. At time 0, a non‐processed sample was taken and the UV lamp was activated. During the UV‐C treatment, 150 ml samples were taken when doses reached 750, 1500, 3000, 6000 and 9000 J l^−1^ (equivalent to 4′47″, 9′51″, 18′54″, 37′34″, 56′00″).

After each UV‐C irradiation dose, 1 mL samples were 10‐fold diluted in peptone water and 0·1 ml inoculated by duplicates onto TBX agar plates (Sigma‐Aldrich) and incubated for 24 h at 37°C. Plates with more than 20 and <300 colonies were counted and results expressed as log_10_ ml^−1^.

### Spray‐drying test

A total of 7 kg of fresh plasma from a commercial manufacturing plant was previously UV‐C treated at 10 000 J l^−1^ prior to inoculation with the *E. coli* strains to eliminate any other bacteria present in the initial raw material. Half amount (3·5 kg) of this UV treated plasma was spiked with the swine *E. coli* K88 isolate at a ratio of 1/47 reaching a final titer of 7·31 ± 0·39 log10 ml^−1^ and the other half with the bovine *E. coli* K99 isolate, at a ratio 1/18 reaching a final titre of 7·66 ± 0·11 log10 ml^−1^. From each of the 3·5 kg inoculated plasma aliquots, two bottles of 750 ml were obtained and spray‐dried in a lab drier (Büchi Mini Spray Dryer B‐290, Büchi Labortechnik, Switzerland) at two different conditions: inlet temperature at 220 ± 1°C and outlet temperature at 80 ± 1°C or 70 ± 1°C, after stabilization of the spray‐drier with water and non‐inoculated control plasma. All studies were performed in triplicate. Air flow through the column was set at 20–27 m^3^ h^−1^ at 20°C. Estimated dwell time was <1 s.

Once SDP was obtained at the two designated outlet temperatures, three tubes containing 0·5 g of dried plasma for each condition were obtained and the dry samples were resuspended in water at a ratio of 1 : 9. From this resuspension, 0·1 ml was seeded in TBX agar for 24 h at 37°C. Colony counting was performed as indicated in the previous section. Results were expressed as a log_10_ g^−1^ of solids according to the equation: log_10_ g^−1^ = log_10_(CFU per ml)/[(% solid content of resuspended sample)/100].

### Modelling of inactivation

The GInaFiT software was used to calculate and plot nonlinear *E. coli* survival curves. The log linear plus tail (Geeraerd *et al*. 2000) and Weibull plus tail (Albert and Mafart 2005) models were tested. The log linear plus tail model (Geeraerd *et al*. 2005) follows the equation (1):

(1) $$\log N=\log\left(\left(10^{\log N0}-10^{\log N_{\mathrm{res}}}\right)\right)-e^{\left(k_{\max}t\right)}+10^{\log N_{\mathrm{res}}}$$

where *k*_max_ is the inactivation rate of the log linear part of the curve; *N*0 is the initial bacterial concentration; *t* is time and *N*_res_ is the number of resistant bacteria sub‐population.

The Weibull model plus tail (Albert and Mafart 2005) uses the equation (2):

(2) $$\log10\left(N\right)=\log10\left(\left(10^{\log N0}-10^{\log N_{\mathrm{res}}}\right)\right)\times 10^{{\left(-\frac{t}{\delta }\right)}^{p}}+10^{\log N_{\mathrm{res}}}$$

where *N*0 is the initial bacterial concentration; *t* is time; *δ* parameter represents the time of the first decimal reduction concentration for the part of the population not belonging to *N*_*res*_; *p* parameter allows to determine concavity or convexity of the curve; and *N*_res_ is the number of resistant bacteria sub‐population.

### Statistical analysis

Data were expressed by means of Log_10_ values and standard deviations of three independent experimental batches. Mean, standard deviations, anova and *F*‐test for comparisons were calculated with Excel 2007 (Microsoft Office). The LSD (Least Significant Difference) test was calculated with Statgraphics Centurion XV ver. 15.2.14 (©StatPoint Technologies Inc, Warrenton, Virginia) to determine significant differences between treatments. Differences at *P *<* *0·05 were considered significant.

Mean square error (MSE), goodness of fit in terms of root mean square error (RMSE), correlation coefficient (*R* ^2^) and adjusted correlation coefficient (adj‐*R* ^2^) values were calculated with the GInaFiT software (Geeraerd *et al*. 2005). The smallest RMSE determined the inactivation model with the best fit (Geeraerd *et al*. 2005).

## Conflict of Interest

Elena Blázquez, Carmen Rodríguez, Jesús Ródenas and Javier Polo are employed by APC Europe, S.L.U. Joy Campbell and Javier Polo are employed by APC Inc. Both companies manufacture and sell spray‐dried animal plasma. Joan Pujols, Ana Pérez de Rozas and Joaquim Segalés declare no conflict of interest.
